# Lactic Acid and Thermal Treatments Trigger the Hydrolysis of *Myo*-Inositol Hexakisphosphate and Modify the Abundance of Lower *Myo*-Inositol Phosphates in Barley (*Hordeum vulgare* L.)

**DOI:** 10.1371/journal.pone.0101166

**Published:** 2014-06-26

**Authors:** Barbara U. Metzler-Zebeli, Kathrin Deckardt, Margit Schollenberger, Markus Rodehutscord, Qendrim Zebeli

**Affiliations:** 1 Institute of Animal Nutrition and Functional Plant Compounds, Department for Farm Animals and Veterinary Public Health, Vetmeduni Vienna, Vienna, Austria; 2 University Clinic for Swine, Department for Farm Animals and Veterinary Public Health, Vetmeduni Vienna, Vienna, Austria; 3 Institute of Animal Nutrition, University of Hohenheim, Stuttgart, Germany; Universidade Federal de Vicosa, Brazil

## Abstract

Barley is an important source of dietary minerals, but it also contains *myo*-inositol hexakisphosphate (InsP_6_) that lowers their absorption. This study evaluated the effects of increasing concentrations (0.5, 1, and 5%, vol/vol) of lactic acid (LA), without or with an additional thermal treatment at 55°C (LA-H), on InsP_6_ hydrolysis, formation of lower phosphorylated myo-inositol phosphates, and changes in chemical composition of barley grain. Increasing LA concentrations and thermal treatment linearly reduced (*P*<0.001) InsP_6_-phosphate (InsP_6_-P) by 0.5 to 1 g compared to the native barley. In particular, treating barley with 5% LA-H was the most efficient treatment to reduce the concentrations of InsP_6_-P, and stimulate the formation of lower phosphorylated myo-inositol phosphates such as *myo*-inositol tetraphosphate (InsP_4_) and *myo*-inositol pentaphosphates (InsP_5_). Also, LA and thermal treatment changed the abundance of InsP_4_ and InsP_5_ isomers with Ins(1,2,5,6)P_4_ and Ins(1,2,3,4,5)P_5_ as the dominating isomers with 5% LA, 1% LA-H and 5% LA-H treatment of barley, resembling to profiles found when microbial 6-phytase is applied. Treating barley with LA at room temperature (22°C) increased the concentration of resistant starch and dietary fiber but lowered those of total starch and crude ash. Interestingly, total phosphorus (P) was only reduced (*P*<0.05) in barley treated with LA-H but not after processing of barley with LA at room temperature. In conclusion, LA and LA-H treatment may be effective processing techniques to reduce InsP_6_ in cereals used in animal feeding with the highest degradation of InsP_6_ at 5% LA-H. Further in vivo studies are warranted to determine the actual intestinal P availability and to assess the impact of changes in nutrient composition of LA treated barley on animal performance.

## Introduction

Barley is an important cereal crop used for livestock feeding and human consumption. It contains relatively large amounts of starch, protein, dietary fiber, and minerals which make this cereal a highly valuable ingredient of the diet [1]. It represents an important source of phosphorus (P), with total P content exceeding 4 g per kg dry matter (DM). However, the availability of P for non-ruminants in barley, like in other cereals and legumes, is low because the major part of P is stored in form of *myo*-inositol hexakisphosphate (InsP_6_) [2], and its salts, also called phytate, serving as a P source for germination [3]. *Myo*-inositol hexakisphosphate is considered an antinutritional factor due to its low digestibility in monogastric animals but also due to its ability to build mineral complexes which inhibit the absorption of cations (e.g., Ca^2+^, Fe^2+^ and Zn^2+^) and protein in the gastrointestinal tract [4], [5]. Endogenous cereal phytases that catalyse the hydrolysis of InsP_6_ to inorganic P and lower *myo*-inositol phosphates (InsP), most importantly *myo*-inositol pentaphosphates (InsP_5_), *myo*-inositol tetraphosphates (InsP_4_), and *myo-*inositol triphosphates (InsP_3_) [6], during germination can be activated by luminal conditions (i.e., pH) in the gastrointestinal tract, rendering a certain amount of P available for the host [7]. Compared with other cereals such as rye and wheat, barley grain possesses lower endogenous phytase activity [2], emphasizing the necessity to treat barley grain to improve intestinal P availability.

Up to now intestinal availability of plant P is mostly enhanced by supplementation of microbial phytases in diets for monogastric livestock species [8], thereby relying on optimal gastrointestinal conditions for maximum phytase activity. Because gastrointestinal pH and digesta passage rate may not always support phytase activity, the degradation of InsP_6_ prior to feeding to animals is of particular interest as lower InsP can be almost completely used by monogastric animals [9]. Traditional processing methods of cereals for human consumption like soaking, malting, germination, and dough fermentation activate endogenous phytase activity thereby promoting the hydrolysis of InsP_6_ [2], [5],[10]–[12]_._ Similar processing techniques may apply in livestock animal nutrition. However, because these processing methods reduce availability and concentration of other nutrients and thus lower the nutritional value, processing of feed (e.g., soaking and fermentation) prior to feeding is mostly restricted to liquid feeding systems for pigs by far [13]–[15]. Lowering pH in the grain stimulates endogenous phytase activity [7]. Therefore, treatment of cereal grains with lactic acid (LA), which is naturally produced during soaking and fermentation in cereal grains, may favor InsP_6_ hydrolysis [5]. Lower concentrations (0.2–0.9%) of LA previously showed to reduce InsP_6_ in barley [16] and may be a suitable processing method to treat barley grain. Also, hydrothermal treatment can reduce InsP_6_ in grains and could therefore lead to a further reduction in InsP_6_ concentration when combined with LA treatment [16]–[18]. Because LA treatment can have additional benefits on health and performance in livestock animals [19]–[24], treatment of barley with LA may be of interest in animal feeding. We hypothesized that soaking barley in increasing concentrations of LA in combination with heat may exert an additive effect on InsP_6_ hydrolyzing properties. The main aim of this study was to evaluate the hydrolyzing capacity of increasing concentrations (0.5, 1 and 5%) of LA alone or in conjunction with heat on InsP_6_ degradation in barley grain and the appearance of intermediate InsP such as InsP_3_, InsP_4_, and InsP_5_, and their respective isomers. We were also interested in the effects of chemical and thermal processing on changes in the overall chemical composition of barley, which might have consequences for the feeding value of barley grain for livestock animals.

## Materials and Methods

### Barley Grain and Lactic Acid

Winter 2-row *Eufora* barley (*Hordeum vulgare* L.) grown during the 2011 season in Eastern Austria was used in this experiment [25]. *Eufora* barley represents a common barley variety used in animal feed and human nutrition in Austria and was provided by the Department of Crop Sciences, Division of Plant Breeding, University of Natural Resources and Life Sciences Vienna, Vienna (research group: H. Grausgruber). After harvesting, grains were carefully cleaned and freed of extraneous matter. Food-grade dl-lactic acid solution (85%, wt/wt) used in this study was purchased from Alfa Aesar GmbH & Co KG (Karlsruhe, Germany). LA solutions (0.5, 1 and 5% LA) were prepared using deionized water (vol/vol). The pH of LA solutions was 2.4, 2.2, and 1.8 for 0.5%, 1% and 5% LA, respectively, prior to treatment.

### Soaking and Thermal Treatment of Grains

The procedure of LA and thermal treatments was the same as described in our previous study [25]. Triplicate barley subsamples were randomly taken and soaked in increasing concentrations of LA, without or with heat treatment (LA-H; only 1% and 5% LA), resulting in an orthogonally designed experiment (i.e., 0.5% LA, 1% LA, 5% LA, 1% LA-H, and 5% LA-H). Based on our previous study [25], where the impact of heat treatment on changes in nutrient composition of barley was small for 0.5% LA, only effects of heat treatment with 1 and 5% LA were investigated in this study. For treatment, a barley subsample (50 g) was soaked in the respective LA solution (1∶1.6 wt/wt) at room temperature (22°C) or heated at 55°C in an oven for 48 hours. Attention was paid that every grain was sufficiently soaked in the treatment solution. After the 48-hours incubation, treated barley samples were spread on Petri dishes and air-dried at 22°C for 24 hours before being ground prior to chemical analysis. Samples of LA-H treatment were cooled to 22°C prior to air-drying. Triplicate subsamples of the untreated *Eufora* barley were used as control (native barley). Only the barley grains were used for subsequent analyses. Drip losses, and thus potential nutrient losses, of the wet barley samples onto the Petri dish were not recovered after air-drying.

### Sample Preparation

Native and dry treated barley samples were ground to pass a 0.5 mm sieve (Type 738, Fritsch, Rudolstadt, Germany). Barley subsamples used for InsP analyses were ground to pass a 0.2 mm sieve, and attention was paid that the ground mass of barley was fine and uniform for analysis. Milled samples were packed in sealed plastic bags and stored at 4°C until further analyses.

### Analyses of Inositol Phosphates

For the analysis of InsP_3_ to InsP_6_ isomers, the ground material was extracted twice with a solution containing 0.2 M EDTA and 0.1 M sodium fluoride (pH 10) using a rotary shaker. Sample to extractant ratio was 1 g to 15 mL, and the total time of extraction was 1 h. After centrifugation the combined supernatants were ultracentrifugated using a Microcon filter (cut-off 30 kDa) devise (Millipore, Bedford, MA, USA) at 14,000×*g* for 30 minutes. Throughout the whole extraction procedure the samples were kept below 5°C. Filtrates were analyzed by high-performance ion chromatography (HPIC) and InsP were detected using a UV detector at 290 nm after postcolumn derivatization using an ICS-3000 system (Dionex, Idstein, Germany) equipped with a Carbo Pac PA 200 column and corresponding guard column. Gradient elution was done with increasing amounts of hydrochloric acid (0.05 M to 0.5 M within 33 minutes). Fe(NO_3_)_3_ solution (0.1% Fe(NO_3_)_3_ × 9 H_2_O in HClO_4_ was used as reagent for derivatization according to Philippy and Bland [26].

InsP_6_ dipotassium salt was obtained from Sigma (Deisenhofen, Germany), InsP_5_ isomers from Sirius Fine Chemicals (Bremen, Germany), InsP_3_ and InsP_4_ isomers, as far as available, were from Santa Cruz Biotechnology (Heidelberg, Germany). These standards were used for peak identification. InsP_6_ was used for calibration. Quantification of lower inositol phosphates was done according to Skoglund et al. [27]. Calibration curves were linear from quantification limit to approximately 10 to 30 µmol/g depending on the InsP isomer.

Quantification limits for InsP-isomers (S/N>10) were 1 µmol/g DM for InsP_3_ and InsP_4_ and 0.5 µmol/g DM for InsP_5_, whereas the detection limits (S/N>5) were 0.5 µmol/g DM for InsP_3_ to InsP_4_ and 0.25 µmol/g DM for InsP_5_. The InsP concentrations were determined as µmol InsP/g DM, and subsequently converted to g P pertaining to each InsP category (i.e., InsP_3_-P, InsP_4_-P, InsP_5_-P, and InsP_6_-P) based on their molecular weight and the respective content of P in the InsP molecule. Samples were analyzed in duplicate. The abundance of the different InsP_3_, InsP_4_ and InsP_5_ isomers was in untreated barley samples as well as in LA and LA-H treated barley was used to evaluate the nature of the InsP_6_ degradation caused by LA and heat treatment.

### Nutrient Analyses

Dry matter, crude ash (CA), crude protein (CP), starch (total, non-resistant (NRS) and resistant starch (RS)), neutral detergent fiber (NDF), and acid detergent fiber (ADF) of the native and treated barleys were determined. Samples were analyzed for DM by oven-drying at 103°C for 4 h [27]. Crude ash was determined by combustion of samples over night at 580°C [28]. Crude protein was analyzed by the Kjeldahl method [28]. The concentrations of NDF and ADF were determined according to official methods [28], [29] using Fiber Therm FT 12 (Gerhardt GmbH & Co. KG, Königswinter, Germany) including heat-stable α-amylase digestion for NDF determination, and were expressed exclusive of residual ash (aNDF_OM_ and ADF_OM_, respectively). The difference between aNDF_OM_ and ADF_OM_ was considered as the hemicelluloses (HC) fraction. For P determination, samples were analyzed using ICP-OES (Vista Pro, Varian, Darmstadt, Germany) after acid digestion using a combination of sulphuric and nitric acid as described previously [30]. Samples were also analyzed for resistant starch (RS) and non-resistant starch (NRS) using a commercial enzymatic RS assay kit (Megazyme International Ireland Ltd., Bray, Ireland) following manufacturer’s protocol, as previously described [25]. Total starch was calculated from RS and NRS fractions. Three subsamples per treatment were analyzed in duplicate.

### Statistical Analysis

Data were subjected to two-way ANOVA using the PROC MIXED of SAS (SAS 9.2, SAS Institute Inc., Cary, NC, USA) with polynomial contrasts between control barley and barley treated with 0.5%, 1% and 5% LA as well as orthogonal contrasts between 1% and 5% LA treatments and 1% and 5% LA-H treatments. Linear patterns were analyzed using contrast statement of SAS accounting for unequal spacing among Control and treatments with 0.5, 1, and 5% LA or Control and treatments 1% LA-H and 5% LA-H. Interactions between LA concentration × heat were assessed where applicable. Duplicates per subsample were averaged and used as the experimental unit in the statistical analysis. Processing method served as fixed effect and sample nested within treatment as random effect. Degrees of freedom were approximated by Kenward-Roger method. Differences at *P*<0.05 level were declared significant.

## Results

### Impact of Lactic Acid and Heat Treatment on the Hydrolysis of Inositol Hexakisphosphate

**Figure 1:**
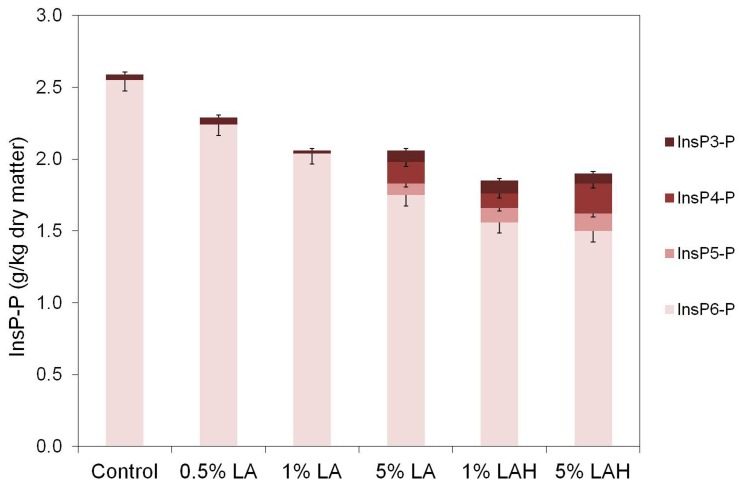
The concentrations of P pertaining to *myo-*inositol tri- to hexakisphosphate (InsP_3_-P, InsP_4_-P, InsP_5_-P, and InsP_6_-P) and to the sum of them (total InsP-P) in untreated barley (control) or barley soaked in increasing concentrations of lactic acid at room temperature in 22°C (LA) or oven-heated at 55°C (LA-H). Data are shown as least square means ± standard error of the mean (n = 3). For InsP_3_-P: all contrasts *P*>0.10; for InsP_4_-P: control vs. LA *P* = 0.50, control vs. LA-H *P* = 0.030, LA vs. LA-H *P* = 0.097; for InsP_5_-P: control vs. LA *P* = 0.10, control vs. LA-H *P*<0.001, LA vs. LA-H *P* = 0.006; for InsP_6_-P: control vs. LA *P*<0.001, control vs. LA-H *P*<0.001, LA vs. LA-H *P*<0.001; for the sum of InsP-P: control vs. LA *P*<0.001, control vs. LA-H *P*<0.001, LA vs. LA-H *P* = 0.055.

The concentration of total P was not different between native and LA treated barley. Additional heat treatment reduced total P concentration by 0.3 g in LA-H treated barley compared to the native barley (Table 1). However, total P concentration did not differ between LA and LA-H treated barley. *Myo*-inositol hexakisphosphate concentration decreased in response to LA treatment and, in particular, when barley was treated with LA and oven-heated at 55°C (Figure 1). Gradual increase in LA concentration from 0 to 5% resulted in a linear decrease in InsP_6_-P concentration from 2.55 g InsP_6_-P/kg DM for control barley to 2.24, 2.04, and 1.75 g InsP_6_-P/kg DM for 0.5, 1, and 5% LA, respectively. The additional heat treatment further lowered the InsP_6_-P concentration to 1.55 and 1.49 g/kg DM for 1 and 5% LA-H, respectively. InsP_3_ was present in all treatments in amounts of 0.05–0.11 g InsP_3_-P/kg DM. InsP_4_ and InsP_5_ isomers were only quantifiable for 5% LA as well as 1 and 5% LA-H, ranging from 0.10–0.14 g InsP_4_-P/kg and 0.16–0.22 g InsP_5_-P/kg DM.

**Table 1:** Nutrient composition of native barley (CON) or barley steeped in various concentrations of lactic acid at room temperature at 22°C (LA) or oven-heated at 55°C (LA-H).

Item^2^	CON	LA			LA-H		SEM^1^	*P*-value^3^		
		0.5%	1%	5%	1%	5%		1	2	3
Dry matter (%)	90.7	93.0	92.2	93.1	91.8	92.2	0.09	<0.001	<0.001	<0.001
Starch (% DM)	59.5	55.2	52.5	54.7	55.3	54.5	0.57	<0.001	<0.001	0.038
Crude protein (% DM)	13.4	13.1	13.6	13.1	13.0	12.9	0.07	0.352	0.004	<0.001
NDF (% DM)	14.5	15.0	16.9	16.8	16.1	12.2	0.30	<0.010	0.490	<0.010
ADF (% DM)	5.34	5.97	6.36	6.20	6.36	5.30	0.220	0.010	0.130	0.110
HC (% DM)	9.15	9.04	10.5	10.6	9.77	6.94	0.360	0.090	0.150	<0.010
Ash (% DM)	2.39	1.98	1.97	1.97	1.90	1.83	0.028	<0.001	<0.001	0.002
P (g/kg DM)	4.11	4.07	3.99	3.85	3.79	3.71	0.084	0.220	<0.010	0.100

1 SEM = standard error of the mean (n = 3).

2 DM = dry matter, NDF = neutral detergent fiber, ADF = acid detergent fiber, HC = hemicelluloses (NDF – ADF), P = total phosphorus.

3 Contrasts, 1 = Control vs. LA, 2 = Control vs. LA-H, 3 = LA (1 and 5%) vs. LA-H (1 and 5%+oven-heating).

**Figure 2:**
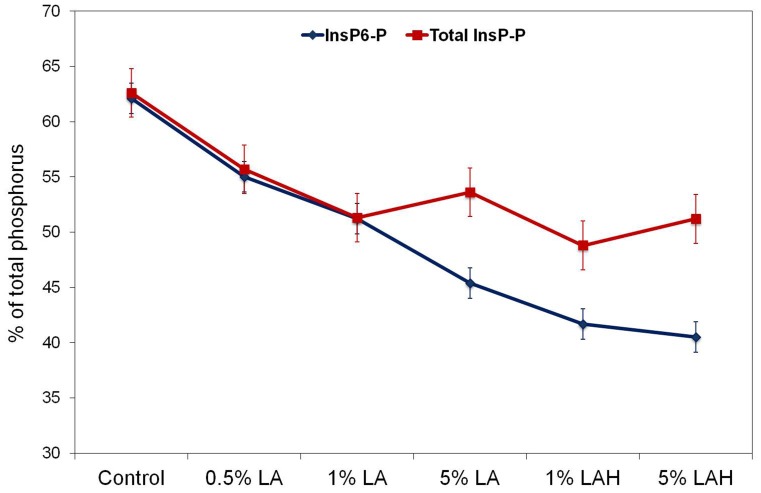
Changes in *myo*-inositol hexakisphosphate (InsP_6_-P) and the sum of InsP_3_-P to InsP_6_-P (total InsP-P) relative to total phosphorus of untreated barley (control) or barley soaked in increasing concentrations of lactic acid at room temperature in 22°C (LA) or oven-heated at 55°C (LA-H). Data are shown as least square means ± standard error of the mean (n = 3). LA and LA-H effects on InsP_6_-P: Control vs. LA *P*<0.01, Control vs. LA-H *P*<0.001, LA vs. LA-H *P* = 0.012; LA and LA-H effects on total InsP-P: Control vs. LA *P*<0.01, Control vs. LA-H *P*<0.001, LA vs. LA-H *P* = 0.14.

Proportions of InsP_6_-P and total InsP-P relative to total P in barley are shown in Figure 2. The InsP_6_-P proportion decreased (*P*<0.01) in barley when treated with LA (from 62.1 in control to 54.7, 50.8, 45.3% for 0.5%, 1% and 5% LA, respectively), and the extent of reduction was greater (*P*<0.001) when the LA-H treatment was applied (41.4 and 40.5% for 1 and 5% LA-H treatments, respectively; Figure 2), compared to the control. Also, when comparing LA-H with LA treated barley, LA-H treatment reduced the proportion of InsP_6_-P compared to LA treatment (*P* = 0.012). The InsP_3_-P was only a very small proportion of total InsP-P in the control barley and barley treated with 0.5% and 1% LA; therefore, total InsP-P mainly comprised InsP_6_-P for these treatments. Due to the increase in InsP_4_-P and InsP_5_-P with 5% LA and 1 and 5% LA-H, the proportion of total InsP-P was similar for LA and LA-H treated barley but lower (*P*<0.01) when compared to the control barley (Figure 2).

**Figure 3:**
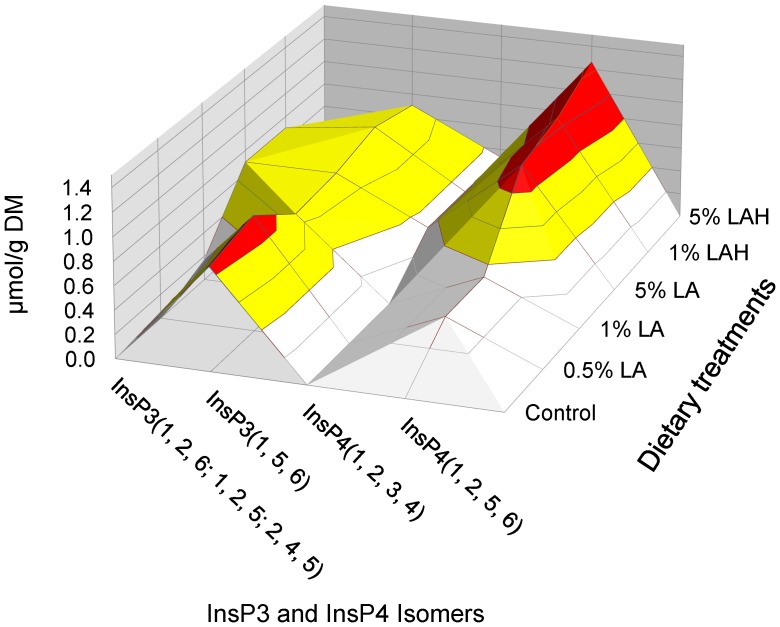
Concentrations of various isomers of *myo-*inositol triphosphate (InsP_3_) and tetraphosphate (InsP_4_) in untreated barley grain (control) or barley grain soaked in increasing concentrations of lactic acid at room temperature in 22°C (LA) or oven-heated at 55°C (LA-H). Data are shown as least square means (n = 3). Isomers exceeding a concentration of 1 µmol/g dry matter were quantified (area labeled in red color); isomers having concentrations between 0.5 to 1 µmol/g dry matter (detection limit and measurement threshold, respectively) were detected but could not be quantified (area labeled in yellow color); are below detection limit of these isomers is shown in white color (<0.5 µmol/g dry matter).

**Figure 4:**
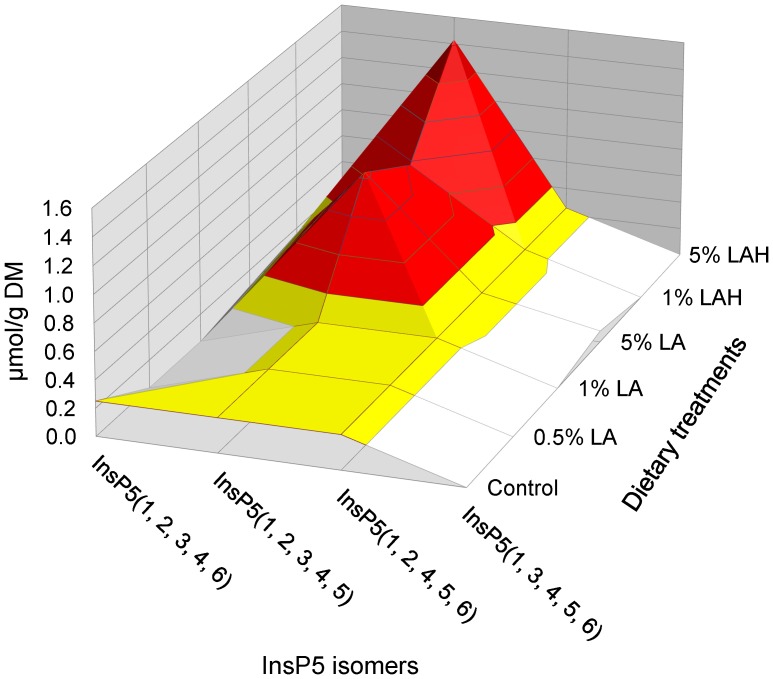
Concentrations of various isomers of *myo-*inositol pentaphosphate (InsP_5_) in untreated barley (control) or barley soaked in increasing concentrations of lactic acid at room temperature in 22°C (LA) or oven-heated at 55°C (LAH). Data are shown as least square means (n = 3). Isomers exceeding a concentration of 0.5 µmol/g dry matter were quantified (area labeled in red color); isomers having a concentration between 0.25 to 0.5 µmol/g dry matter (detection limit and measurement threshold, respectively) were detected but could not be quantified (area labeled in yellow); area below detection limit of these isomers is shown in white color (<0.25 µmol/g dry matter).

The Ins(1,5,6)P_3_ was quantifiable for the control barley and all treatments. In the control group as well as in the treatment with 0.5% LA a peak deriving from one or more of the coeluting isomers Ins(1,2,6)P_3_, Ins(1,4,5)P_3_ and Ins(2,4,5)P_3_ was not detectable, whereas a peak from coeluting isomers was detectable but not quantifiable in barley treated with 5% LA and 1 and 5% LA-H (Figure 3). The Ins(1,2,5,6)P_4_ was the predominant InsP_4_ isomer in barley treated with 5% LA and 1 and 5% LA-H (Figure 3). The InsP_4_ isomer was found in barley treated with 0.5 and 1% LA in amounts below the quantification limit but was not detected in control barley. Furthermore, Ins(1,2,3,4)P4 was found in 5% LA and 1 and 5% LA-H treated barley but not in the other treatments. In 5% LA and 1 and 5% LA-H treated barley, Ins(1,2,3,4,5)P_5_ was the primary InsP_5_ isomer. Ins(1,2,3,4,6)P_5_ was detected in the control group and in LA-H treatments, Ins(1,2,4,5,6)P_5_ were above the detection limit but not quantifiable for all treatments (Figure 4).

### Impact of Chemical and Heat Treatment on Barley’s Chemical Composition

After soaking barley for 48 h, pH values of barley treated with LA or LA-H raised by 0.3 to 1.2 units (pH 2.5, 2.7, 2.1, 2.7 and 2.1 for 0.5% LA, 1% LA, 5% LA, 1% LA-H and 5% LA-H pre-incubation; and pH 3.7, 3.2, 2.4, 3.9 and 3.0 for 0.5% LA, 1% LA, 5% LA, 1% LA-H and 5% LA-H after 48 h of incubation, respectively).

**Figure 5:**
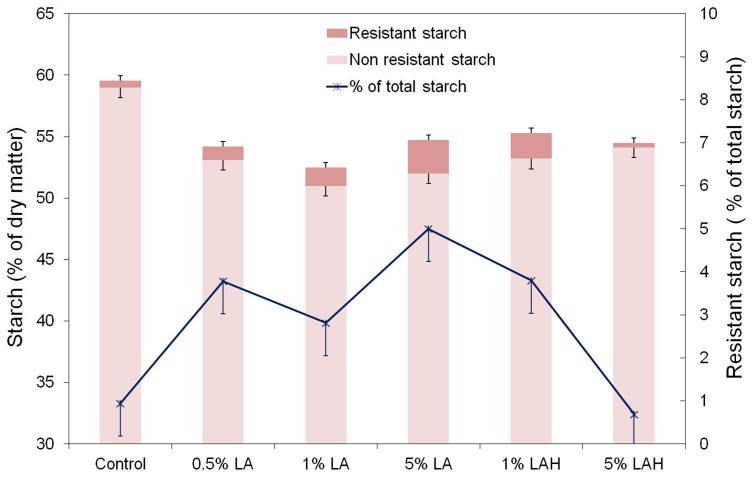
Changes in concentrations of resistant starch (RS) and non-resistant starch (NRS) of untreated barley (Control) or barley soaked in increasing concentrations of lactic acid at room temperature in 22°C (LA) or oven-heated at 55°C (LA-H). Data are shown as least square means ± standard error of the mean (n = 3). LA and LA-H effects on RS: Control vs. LA *P* = 0.006, Control vs. LA-H *P* = 0.202, LA vs. LA-H *P* = 0.049; LA and LA-H effects on RS relative to total starch: Control vs. LA *P* = 0.006, Control vs. LA-H *P* = 0.186, LA vs. LA-H *P* = 0.051; LA and LA-H effects on NRS: Control vs. LA *P*<0.001, Control vs. LA-H *P*<0.001, LA vs. LA-H *P* = 0.033.

The greatest change in the chemical composition was observed for the starch content when comparing treated barley samples with the native barley grain (Table 1, Figure 5). In general, LA and in particular LA-H treatment decreased (*P*<0.05) total starch content of barley. Resistant starch, both as g/kg DM and as proportion of total starch, was higher (*P*<0.001) in LA treated barley than in control barley (Figure 5) and peak increase was attained by 5% LA (RS relative to total starch: 0.9 in control vs. 5% in 5% LA). However, when the barley samples that were treated with 5% LA underwent thermal treatment, RS content was comparable to the native barley grain (Figure 5).

The CP content of barley did not change when barley was treated with LA, but additional heat treatment lowered the concentration of CP by 0.4% units compared to the control barley (Table 1). Moreover, LA and LA-H treatment modified the fiber fractions of barley. The contents of aNDF_OM_ and ADF_OM_ increased by 1.7 and 0.8% in response to LA treatment, respectively, whereas the content of HC remained similar for LA treated and control barley. The heat treatment increased aNDF_OM_ and ADF_OM_ concentrations in barley when 1% LA treatment was used compared to the control, whereas heat decreased the aNDF_OM_ concentration by approximately 2% when barley was soaked in 5% LA (Table 1). This finding suggests an interaction (*P*<0.01) between LA and heat treatment for these variables. As a consequence, HC content was reduced by approximately 2.5% with the 5% LA-H treatment compared to the control barley. Barley treated with LA and LA-H also contained less crude ash than the native control barley.

## Discussion

There is an increasing interest in enhancing utilization of minerals from cereal grains used in animal nutrition. This strategy alleviates the dependency on inclusion of large amounts of inorganic P in animal diets with great economical and ecological importance [8], [31]. Because of the low availability of P in cereals for monogastric livestock species, a range of feed processing techniques has been applied to reduce their InsP_6_ concentration. Yet, in the feeding of monogastric livestock species such as swine and poultry, processing techniques used to increase P availability of feeds are often restricted to microbial phytase supplementation [8], [32]. Our data indicated that treatment of barley grain with LA and LA-H was able to decrease the InsP_6_ concentration and thus potentially increase P availability in barley. Most previous studies investigating the effect of LA on phytate degradation focused only on InsP_6_ disappearance [16], [18]. Here, we could show characteristic changes in the accumulation of lower InsP, such as InsP_3_ to InsP_5_, related to the LA concentration and heat treatment. These lower InsP may interfere less in intestinal mineral availability than InsP_6_; however, InsP_3_ to InsP_5_ still bind P and can have an inhibitory effect on mineral absorption [33]. Because soaking of cereals in water is current practice in liquid feeding systems for livestock, we abstained from comparing the effects of LA treatment with soaking barley in water in the present study. Also, the present processing of barley grain aimed at being applied in dry feeding systems; therefore, the comparison between the native barley and the LA-treated barley was more relevant for the present study than the comparison between soaking in water and LA.

Overall, the concentration of total P and InsP_6_ in native barley were in accordance with data from previous studies [34]–[37] showing comparable InsP_6_ disappearances when barley was treated with LA and and LA-H [16], [18], [38]. Accordingly, the InsP_6_ reducing effect of LA was more pronounced at higher concentrations and potentiated by the heat treatment [16], [18], [38]. The most effective treatments in the present study, i.e. 5% LA, 1% LA-H and 5% LA-H, converted 17 to 22% of InsP_6_-P into inorganic P or lower InsP-P in barley grain and the disappearance of InsP_6_-P was about 10% greater with heat treatment than at room temperature.

Plant phytases and InsP_6_ are mostly localized in the aleurone layer of cereal grains [39]–[41]. The two phytases isolated from barley are activated in wet conditions when a slightly acidic pH of 5 and 6 is reached, respectively [39]. Soaking of cereal grains stimulates endogenous LA production causing lower pH with progressing incubation time [16], [36], [40], [42]. Treating barley grains with LA solutions might therefore mimic the endogenous LA production, shortening the time until the critical pH value is reached for phytase activation. However, in this experiment pH values of LA treated barley were much more acidic than the actual pH values for optimum endogenous phytase activity. Possible explanations for InsP_6_ removal during treatment with LA without or with heat may therefore be that endogenous phytases of barley may have been shortly activated during the soaking process and a certain phytase activity during the drying process cannot be excluded, thereby contributing to the inorganic P release with LA and LA-H treatment. Yet, a reduction in phytase activity was previously found in barley grains soaked in 0.8% LA when compared to barley soaked in water after 48 and 96 h of incubation [16]. Endogenous phytase activity was not determined in the present study. However, it can be assumed that other processes, such as leaching of nutrients and acidic ester hydrolysis, than an enhancement of phytase activity likely contributed to the InsP_6_ degradation in the present study. Soaking processes are generally associated with leaching of nutrients including minerals [3], [31], [43]. Leaching of minerals into the soaking medium may have been indicated by the lower crude ash concentration in treated barley samples and the higher pH of the soaking medium after the 48-hour incubation compared with initial pH values. Heat treatment can potentiate the soaking effect as the heat causes structural changes in the grain leading to a more rapid hydration (i.e. swelling) of the grain [31], [40], [44]. As we could only observe a reduction in total P of barley when treated with 1 and 5% LA-H, loss of P and with this of InsP_6_ by leaching may have been mostly restricted to these treatments. Haraldsson and coworkers [16] estimated that a loss of 5% of InsP_6_ during soaking and heat treatment (48°C) of barley with 0.8% LA could be explained by leaching processes in their study.

Another explanation for the reduction in InsP_6_ in response to LA and LA-H treatment of barley could be related to the low pH in the soaking medium. Phosphate groups are esterified to the inositol ring of InsP, and can be removed by acidic ester hydrolysis [16], [31], [45]. Our data suggest an acceleration of acid hydrolysis of InsP_6_ in response to additional heat treatment, which is indicated by the lower InsP_6_ concentration and the accumulation of InsP_4_ and InsP_5_ for LA-H treated barley. Because only small amounts of InsP_5_ to InsP_3_ were detected, it is likely that this treatment might have triggered a complete degradation of lower InsP as soon as the first phosphate group was released from InsP_6_ [44]. The accumulation pattern of lower InsP isomers may help to differentiate whether InsP_6_ hydrolysis was more related to endogenous phytase activity or pH and heat. In this experiment, the occurrence of Ins(1,2,3,4,5)P_5_, Ins(1,2,3,4)P_4_, Ins(1,2,5,6)P_4_ and Ins(1,2,6)P_3_ with 5% LA and 1 and 5% LA-H may indicate the action of cereal phytases because these phytases, like barley phytases P1 and P2, are suggested to be 6-phytases [E.C.3.1.3.26] [35], [46], [47]. However, an ultimate distinction between endogenous 6-phytase action and pH and heat effects cannot be made using the present experimental design.

In line with previous studies evaluating soaking procedures [43], the LA and LA-H treatment of barley resulted in small losses of other nutrients. Observed changes in nutrient composition may reduce the feed value of LA and LA-H treated barley, with the decrease in total starch as the most critical loss for the feed value as it affects the energy concentration of barley. Despite its indigestibility for the host animal, the greater RS concentration of LA treated barley may increase the functional and thus health-promoting potential of barley for livestock animals, such as pigs [48] and ruminants [19]–[22], [49]. Aside from leaching of nutrients, e.g. minerals, starch, and water-soluble protein into the soaking medium and potentiation of this effect by heat treatment [43], it is thinkable that the low pH in the soaking medium modified the molecule structure of some nutrients; for instance leading to the higher RS content of barley with increasing LA concentration [25]. Interestingly, the combination of the highest LA concentration and heat likely abolished the effect on RS formation, which confirms previous findings [25].

The leaching of certain nutrients into the soaking medium likely caused an increase in concentrations of other nutrients in barley such as fiber fractions. Here, aNDF_OM_ and ADF_OM_ contents increased for all LA and 1% LA-H treated barley samples thereby maintaining a similar HC content among treatments. Yet, low pH combined with heat treatment seemed to catalyze degradation of fibrous components in barley grain as indicated by the lower aNDF_OM_, ADF_OM_ and HC contents for 5% LA-H treatment compared to all LA and 1% LA-H treatments. Fibrous components can be mostly found in the three aleurone layers of the barley grain and mainly consist of cellulose, arabinoxylan and mixed-linked β-glucan [50]. According to previous studies, the arabinoxylan fraction may be more susceptible to low pH and heat than the cellulose and β-glucan fractions [16], [51], [52]. The β-glucan fraction in barley may even be stabilized by LA and heat treatment [16].

Finally, the total InsP_6_ degradation by LA and LA-H treatment may remain below the degradation extent reported by dietary supplementation of microbial phytase [34]. Yet, the conditions for the pre-treatment of barley grain may be more easily controlled and stabilized than luminal conditions in the gastrointestinal tract which are necessary to guarantee sufficient InsP_6_ degradation. Optimum microbial or cereal phytase activity depends on gastrointestinal pH and passage rate and may be biased in case luminal conditions are suboptimal in vivo. For instance, in pigs after weaning gastric pH may not reach the necessary acidic pH for microbial phytase activation [53]. Even in cattle nutrition, the inclusion of phytases has been suggested to optimize the utilization of dietary P [54] which indicates that, despite the highly complex rumen microbiota and long ruminal retention times of about 30–48 hours of ingested feed [55], InsP_6_ hydrolyzing capacity may be limited, in particular when short forage particle size is fed [54]. Additional advantages of LA treatment of barley are improved storage stability by decreasing molding of the grain post-harvest and, when eaten, support of gastric barrier function in monogastric livestock animals [56]. In dairy cows, treating the barley fraction of the diet with 0.5 and 1% LA proved beneficial for rumen fermentation and immune-metabolic health status of the animals [19]–[22].

In conclusion, treating barley grain with LA or LA-H may be effective processing techniques to reduce the InsP_6_ concentration of cereals used in animal feeding. The greatest InsP_6_ hydrolysis was observed with the highest investigated LA concentration of 5% and heat treatment. Lower InsP profiles obtained with 5% LA, 1% LA-H and 5% LA-H treatments are similar to profiles found when microbial 6-phytase is applied. Changes in nutrient composition of barley grain due to LA and LA-H treatment, i.e. lower starch and ash concentrations and accumulation of fiber fractions, may impact the feed value of barley, but might increase its health-enhancing properties, in particular, due to greater concentrations of RS and dietary fiber. To determine the actual intestinal P availability and to assess the effects of changes in nutrient composition and functional abilities of LA and LA-H treated barley, further in vivo studies are needed.
